# Acute cannabidiol treatment enhances social interaction in adult male mice

**DOI:** 10.3389/adar.2023.11163

**Published:** 2023-05-10

**Authors:** Livia F. Ferreira, Nikhita Pathapati, Stephen T. Schultz, Mary C. Nunn, Bethany L. Pierce, Yatzil R. Sanchez, Meredith D. Murrell, Brett C. Ginsburg, Emmanuel S. Onaivi, Georgianna G. Gould

**Affiliations:** ^1^ Center for Biomedical Neuroscience, The University of Texas Health Science Center at San Antonio, San Antonio, TX, United States; ^2^ Department of Cellular and Integrative Physiology, School of Medicine, The University of Texas Health Science Center at San Antonio, San Antonio, TX, United States; ^3^ Biological Psychiatry Analytic Laboratory, The University of Texas Health Science Center at San Antonio, San Antonio, TX, United States; ^4^ Department of Psychiatry and Behavioral Sciences, The University of Texas Health Science Center at San Antonio, San Antonio, TX, United States; ^5^ Cannabis Research Institute, William Paterson University, Wayne, NJ, United States; ^6^ Department of Biology, William Paterson University, Wayne, NJ, United States

**Keywords:** CBD, serum metabolites, social preference, marble burying, social dominance

## Abstract

Cannabidiol (CBD) is a non-intoxicating phytochemical from *Cannabis sativa* that is increasingly used to manage pain. The potential for CBD to ameliorate dimensional behavior symptoms occurring in multiple psychiatric disorders was suggested, including social interaction impairments. To test this hypothesis, adult male BTBRT+Itpr3tf/J (BTBR) mice, a model of idiopathic autism exhibiting social preference deficits and restrictive repetitive behaviors, were acutely treated with vehicle or 0.1, 1, or 10 mg/kg CBD. Social interaction preference was assessed 50 min after treatment, followed by social novelty preference at 60 min, marble burying at 75 min and social dominance at 120 min. CBD (10 mg/kg) enhanced BTBR social interaction but not social novelty preference, marble burying or dominance, with serum levels = 29 ± 11 ng/mg at 3 h post-injection. Next, acute 10 mg/kg CBD was compared to vehicle treatment in male serotonin transporter (SERT) knock-out mice, since SERT deficiency is an autism risk factor, and in their wildtype background strain controls C57BL/6J mice. CBD treatment generally enhanced social interaction preference and attenuated social novelty preference, yet neither marble burying nor dominance was affected. These findings show acute treatment with as little as 10 mg/kg purified CBD can enhance social interaction preference in male mice that are otherwise socially deficient.

## Graphical Abstract

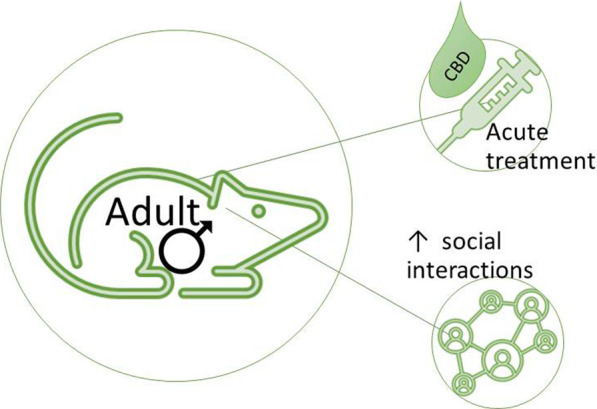

## Introduction

Cannabidiol (CBD) is a bioactive, non-psychotomimetic isolate from *Cannabis sativa* (hemp or marijuana) that occurs naturally in several derivative forms (1). Most bioactive compounds from marijuana are schedule I substances (illegal, high risk of abuse) in the United States of America unless proven to contain <0.3% of the psychotrophic Δ-9-tetrahydrocannabidiol (THC) (2). However, a purified CBD product, Epidiolex, was approved for treatment of severe epilepsy in 2018 by the United States Food and Drug Administration (FDA) as a low abuse risk schedule V drug (3, 4). Yet essential data on preclinical efficacy, pharmacokinetic and pharmacodynamic of CBD and other non-psychoactive cannabinoids to guide medical use remains scarce (5). The potential of CBD as a therapeutic for autism was suggested (6), but there are not enough reports on its efficacy, actions or adverse effects in humans or animal models are available to assess its utility for treating core behavior symptoms (7, 8).

CBD interactions with cannabinoid (CB_1_ and CB_2_) receptors are complicated: Based on agonist interactions, CBD was initially considered a potent antagonist or inverse agonist of CB_1_ and CB_2_ receptors (9). However, relative to THC, CBD has low affinity for both CB receptors (10). Instead, CBD may act as a CB receptor allosteric modulator (11). Also, CBD interacts with receptors such as GPR55, serotonin 5-HT_1A_, adenosine A_2A_ and ion channels such as the transient receptor potential vanilloid1 (TRPV1 or capsaicin) channel and its related TRPV isoforms to modulate sensations, behavior, and immune function (2, 12). Finally, CBD may slow the synaptic clearance of endogenous cannabinoids, since it inhibited fatty acid amide hydrolase (FAAH) in rat brain more potently (IC50 = 15.2 ± 3.2 µM) than any other cannabinoid tested (13).

Several CBD preclinical studies generally support the idea it might ameliorate autism symptoms. For example, in mice, CBD treatment had anxiolytic properties, reducing time in closed arms of the elevated plus maze (14). CBD also attenuated aggression in socially isolated mice (15). The most treatment-resistant autism symptom is social behavior impairment; yet reports of acute CBD effects in three-chamber social preference tests (16) for inbred strains used in drug screens were lacking.

The main goal of this initial study was to test the hypothesis that CBD administration can ameliorate autism-like behaviors in two mouse models with established behavioral face validity to autism. Both the BTBR T+Itpr3tf/J (BTBR) strain (17, 18) and serotonin transporter (SERT) knock-out mice (19, 20) exhibit impaired behaviors paralleling characteristic autism deficits. As a treatment control, the behavioral responses of normally gregarious C57BL/6J mice to acute CBD treatment were also measured. Since autism is four times more prevalent in males, and autism behavior deficits occur throughout the lifespan, adult male mice were tested (21). Our goal was to find the lowest dose of pure CBD that could enhance social interaction preference, social dominance, or reduce restrictive-repetitive traits such as marble burying in male mice.

## Materials and methods

### Animals

Subjects were 44 adult male (4–6 month old) BTBR (BTBR T+Itpr3tf/J, JAX strain stock # 002282, 9–10 mice/treatment, 4 for a pilot 50 mg/kg CBD dose test) with (mean ± SEM) weight = 27 ± 1 g, and 20 C57BL/6J (JAX strain stock #000664) with weight = 29 ± 1 g. They were progeny of mice purchased from Jackson Laboratories (Bar Harbor, ME, USA) bred in-house for >5 generations. Sixteen male serotonin transporter (SERT) knock-out mice (22), 4 months old, with weight = 28 ± 1 g, were a gift from Dr. Lyn Daws. Genotypes of SERT knock-out mice were confirmed from tail biopsies collected after euthanasia by Transnetyx (Cordova, TN, USA). Stimulus (stranger) mice for sociability and dominance tests were age matched 129S1/SvImJ (JAX strain stock # 002448), with (mean ± SEM) weight = 27 ± 1 g, also bred in house.

The housing room had a 14:10 h light–dark cycle, with lights (300 lux, measured by Lux Light Meter Pro App for iOS) on at 0700 h, and temperature of 22°C–25°C. Mice were kept in sterilized JAG75 cages (29 × 18 × 12 cm) with wood-chip bedding (Teklad, Harlan, Indianapolis, IN), but no enrichment items in ventilated housing racks (Micro-Vent 140-cage mouse IVC model for JAG75, 60 air cages/h from Allentown, Inc., Allentown, PA, USA). Stimulus mice were kept on a separate, non-facing housing rack from the subjects. Mice had *ad libitum* access to chow (Teklad #7912, Harlan, Madison, WI) and water (reverse-osmosis and acidified to pH 2.5–3.0 using HCl) and were housed 4 to 5 per cage with same-sex and strain cage-mates born within a week of each other. All mice were euthanized by cervical dislocation and decapitation by an experienced researcher 3 h after behavior tests. Procedures involving live mice complied with ARRIVE guidelines (23), the updated US Guide for the Care and Use of Laboratory Animals (24) and were approved by the University of Texas Health Science Center at San Antonio Institutional Animal Care and Use Committee.

### Acute cannabidiol treatments

**FIGURE 1:**
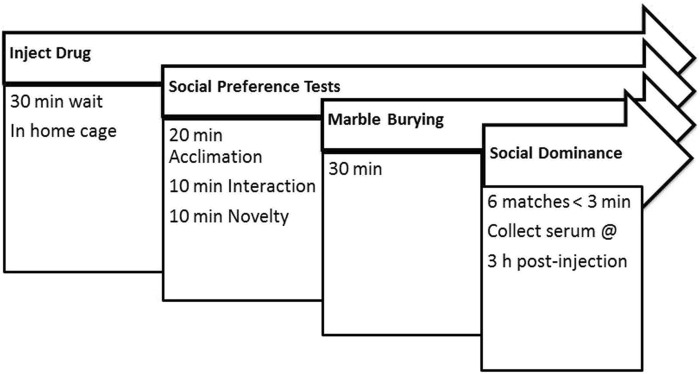
Experimental timeline. Mice were treated with a single dose of cannabidiol or vehicle and were tested on the same day in all behaviors. At 3 h after administration and 30 min after the last dominance test, mice were euthanized and serum collected for measurement of CBD and its metabolites.

An hour prior to treatment, mice were moved in home cages from their housing room to the behavior testing room, dimly lit (16 lux) with red compact fluorescent light bulbs. Mice were weighed on a digital scale (Taylor 3804, Office Depot, USA), and within each cage mice were injected (i.p. volume 0.1 mL/10 g) with either freshly made control vehicle (10% methanol in saline), 0.1, 1, or 10 mg/kg CBD (diluted from catalog #90081, cannabidiol USDEA exempt preparation of 10 mg in 1 mL methanol, Cayman Chemical Co., Ann Arbor, MI, USA) 30 min prior to three chamber test arena conditioning and 50 min before the first phase of social preference tests. The treatment and tissue collection time course of the present study was chosen since the half-life of systemic CBD administration was 3.9 h in mice (25). Permanent marker lines used on tails identified each mouse, and mice were returned to home cages for 30 min so CBD treatments could begin to take effect. The experimental timeline for behavior testing of all mice in the study is shown in Figure 1. A pilot test of 4 BTBR mice dosed with 50 mg/kg CBD in 50% methanol/saline solution was needed to achieve this concentration of CBD, however the methanol also impaired their locomotor activity too much to use in behavior tests, but their serum and brain was used for analytical biochemistry measurements.

### Sociability preference tests

Three chamber tests for social interaction and social novelty preference were performed as previously described (16, 26). Age matched male 129S1/SvImJ mice were pre-conditioned to be stimulus strangers in interaction tests *via* 3 daily confinements, each lasting 30 min, under wire mesh cups before their use in tests. To acclimate, under low red light (16 lux) each subject mouse was placed alone in the middle of a 3-chamber test arena for 10 min. Then interior doors were opened so subjects could explore the full arena for 10 min, and we observed them enter both end chambers at least once as criterion for use in tests. Two BTBR mice treated with 0.1 and 1 mg/kg CBD were dropped from the experiment for failure to enter all three chambers, so sample size in these groups became 9 instead of 10 mice. Social interaction tests began after wire cup cages, one empty (novel object) and the other holding a same-aged stranger male mouse, were randomly placed in opposite end chambers. Mice in arenas were video recorded for 10 min. To measure social novelty preference, subjects were confined in the center chamber as a new stranger mouse was introduced under the empty cage and the “old” stranger in the cage was moved slightly. Doors were opened for subjects and mice were video recorded for another 10 min. Tests took place between 1,300 and 1,500 h, and 6 mice were tested together in different arenas at once.

Data from mouse videos was collected by treatment blind observers. The cumulative time each mouse spent in chambers with strangers (old and new) or objects (cages) and sniffing them was measured and compared within and between groups. The number of times subject mice entered different chambers, time spent in middle chambers, and the number of fecal boli in the middle chamber were also measured.

### Marble burying

After sociability tests, subjects were placed individually in 40 cm × 20 cm sterilized cages filled with wood chip bedding, 8 cm deep, on which 15 flat blue glass marbles were put in a 3 × 5 grid. Room lighting was 16 lux. The number of marbles with surface area >2/3 buried with bedding was tallied after 30 min by treatment blind observers.

### Social dominance

Tube tests for social dominance were performed as previously described (27) by researchers blind to the treatments. Tubes were clear acrylic 3 cm ID and 31 cm length with a central slot and removable mesh divider, room lighting was 300 lux. Male mice were placed in tube test matches against 6 age and sex matched 129S1/SvImJ mice, which were previously conditioned in tube tests against each other. Mice were inserted nose to nose in the tube. As a timer was started the middle insert was removed. The first mouse to advance causing the partner to back out was the match winner, got 1 point, while the mouse that backed out was the loser, got 0 points. Matches exceeding 3 min were a tie, so each mouse got 0.5 points.

### Measurement of Serum and brain CBD and metabolites by HPLC-tandem MS

CBD and its metabolites 7-OH-CBD, and 7-COOH-CBD, along with the internal standard deuterated CBD-D3 were obtained from Sigma-Aldrich Chemical (St. Louis, MO). The CBD metabolite 6-α-OH-CBD was obtained from Cayman Chemical (Ann Arbor, MI). HPLC grade methanol, isopropanol, acetonitrile and hexane were purchased from Fisher (Fair Lawn, NJ). All other reagents were purchased from Sigma Chemical Company (St. Louis, MO).

Milli-Q water was used for preparation of all solutions. CBD, metabolites, and CBD internal standard super stock solutions were prepared in methanol at a concentration of 1 mg/mL and stored in aliquots at −80°C. Working stock solutions were prepared each day from the super stock solutions at concentrations of 0.1, 1, and 10 μg/mL were used to spike the calibrators.

CBD, and metabolites, were quantified in mouse serum samples and whole brain homogenates using HPLC with tandem mass spectrographic detection. Briefly, 100 µL of plasma for spiked calibrators and unknown serum samples were mixed with 500 µL of isopropanol by vortexing vigorously with 20 µL of 10 μg/mL of deuterated CBD (internal standard). Hexane (1 mL) was then added to each sample and vortexed for 30 s. The samples were then centrifuged for 10 min at 32K g, and the supernatants transferred to 1.5 mL microcentrifuge tubes and dried under a nitrogen stream. The residue was resuspended in 50 µL of mobile phase A (10 mM ammonium formate 100% HPLC grade methanol, 0.1% formic acid) and 10 µL were injected into the LC/MS/MS. The ratio of the peak area of CBD and metabolites to CBD-D3 internal standard was compared against a linear regression of calibrator response ratios at concentrations of 0, 5, 10, 50, 100, 500, and 1,000 ng/mL of each in control plasma to quantify CBD and metabolites. The concentration of CBD and metabolites was expressed as ng of each compound/mL serum. Samples were identified as positive if any of the precursor or metabolites were detected above the lower limit of quantitation, determined to be 5 ng/mL.

The HPLC system consisted of a Shimadzu CBM-20A Controller, LC-20AD pumps, SIL-20AC autosampler, and an AB Sciex API 4000 tandem mass spectrometer with turbo ion spray using positive mode. The analytical column was an ACE 3 µm C18 75 × 3.0 mm (Mac Mod- Chaddsford, PA) and was maintained at room temperature (27°C) during the chromatographic runs. The flow rate of the mobile phase was 0.3 mL/min. CBD and metabolites were eluted with a step gradient. The column was equilibrated with 100% mobile phase A. At 6.1 min after injection, the system was switched to 100% mobile phase B (10 mM ammonium formate and 0.1% formic acid dissolved in 90% HPLC grade methanol). At 8.1 min, the system was switched back to 100% mobile phase A. LC-ESIMS/MS analysis using selected reaction monitoring was used to detect and quantify the major fragments of CBD and its metabolites. Analyst 1.1 software (Applied Biosystems) was used to identify the following transitions to quantify CBD and metabolites: CBD 315.315→193.3 Da (CBD), 331→201 (7-OH-CBD), 345→299.3 (7-COOH-CBD), 331.2→271.1 (6-alpha-OH-CBD), 318.1→196.3 (CBD-D3).

### Statistical analyses

Social interaction and social novelty test data from BTBR mice treated with different doses of CBD were compared by two-way analysis of variance (ANOVA) for time in chamber and sniffing choice tests, with Sidak’s post-hoc test to adjust for multiple comparisons of any significant outcomes within and between groups. Preference test time differences, chamber entries, and middle dwelling between groups, mean number of feces, buried marbles, and tube test times were compared by one-way-ANOVA and Sidak’s post-hoc tests. For wins in dominance tube tests, since possible outcomes of 0 = loss, 0.5 = draw or 1 = 1 are non-parametric, a Kruskal-Wallis ANOVA or Mann-Whitney U test was performed. For C57BL/6J and SERT knock-out mice social interaction and novelty test data were compared using a mixed effects model (REML) ANOVA, and all other parameters were compared by unpaired two-tailed t-tests. Prism (version 9, GraphPad, LaJolla, CA, USA) was used for all statistics and graphs. Data are shown as mean ±95% confidence interval (95% CI) in graphs and reported as mean ± standard error of the mean (X̅ ± SEM) in the text, unless otherwise noted.

## Results

### Social preferences

#### Dose-dependent effect of cannabidiol on BTBR social interaction preference

**FIGURE 2:**
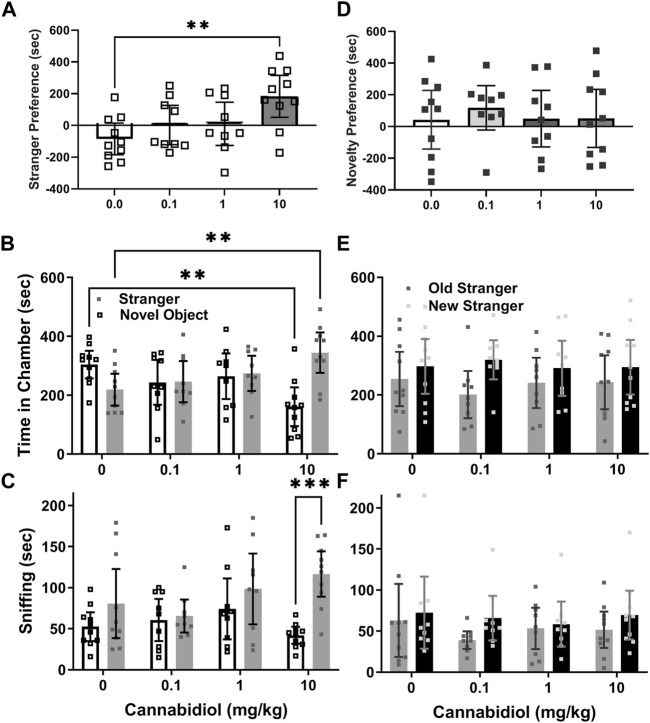
Acute cannabidiol treatment enhances social interaction preference in adult male BTBR mice. **(A)** Cannabidiol (CBD) at 10 mg/kg reduced time spent in chambers with novel objects (**p < 0.005) and increased (**p < 0.005) time spent in chambers with stranger mice relative to vehicle control mice. **(B)** By measure of sniffing time, cannabidiol (10 mg/kg) enhanced sniffing of stranger mice versus novel objects (***p < 0.0005) based on within-group comparisons. **(C)** In social novelty tests there were no effects of cannabidiol treatment on time spent in the stranger chambers. While BTBR mice generally preferred novel over familiar stranger chambers, within the groups there were no significant preferences. **(D)** By measurements of **(E)** time in chambers or **(F)** of sniffing, there was no preference for social novelty. Samples sizes were vehicle = 10, 0.1 mg/kg = 9, 1 mg/kg = 9, 10 mg/kg = 10 mice; data are mean ±95% CI, small squares are individual values.

The social interaction preference, or difference between stranger and novel object chamber time, for BTBR males was greater than vehicle controls in the 10 mg/kg CBD group (F (3, 34) = 4.6, *p* = 0.009, Sidak’s t (34) = 3.6, *p* = 0.003), as shown in Figure 2A. The two-way ANOVA interaction was significant, as BTBR males treated with 10 mg/kg CBD spent less time in novel object chambers (dose x chamber F (3, 68) = 8.2, *p* = 0.0001, Sidak’s t (68) = 3.6, *p* = 0.002), and more time in stranger chambers (t(68) = 3.2, *p* = 0.006) than controls, as shown in Figure 2B. In multiple comparisons within groups only 10 mg/kg treated BTBR preferred stranger mice over novel objects (t(68) = 4.7, *p* < 0.0001).

The sniffing time preference, or difference in sniffing time between strangers and novel objects for BTBR males approached, but was not significantly greater for the CBD 10 mg/kg group than its vehicle control group (F (3, 34) = 3.15, *p* = 0.037, Sidak’s t(34) = 2.41, *p* = 0.06). The two-way interaction was not significant (dose x chamber F (3, 68) = 2.6, *p* = 0.06). However, the chamber side effect was significant, and within group comparisons revealed that time spent sniffing stranger mice was longer than the time sniffing novel objects (chamber F (1, 68) = 12.8, *p* = 0.001, Sidak’s t(68) = 4.15, *p* = 0.0004) only for the 10 mg/kg CBD treated group (Figure 2C).

#### No effect of cannabidiol on BTBR social novelty preferences

In social novelty preference tests, novelty preference, or difference in time spent in novel versus familiar stranger chambers was not significant between CBD dose groups (F (3, 34) = 0.21, *p* = 0.89, Figure 2D). The 2-way ANOVA revealed a chamber side effect (F (1, 68) = 5.71, *p* = 0.02), but no interaction or CBD dose effect for social novelty preference, based on time in chambers (Figure 2E). For social novelty sniffing time, no preference differences between novel versus familiar strangers emerged for any CBD dose (F (3, 34) = 1.6, *p* = 0.21). The two-way ANOVA also indicated no interactions, or differences among CBD doses or chamber side times for sniffing preferences of any treatment groups (Figure 2F).

#### Other BTBR behaviors during social preference tests

Middle chamber dwelling time was compared across CBD doses, and there were no significant differences during social interaction (F(3, 34) = 1.3, *p* = 0.30, pooled X̅ ± SEM = 86 ± 9 s) or social novelty preference tests (F(3, 34) = 1.1, *p* = 0.34, pooled X̅ ± SEM = 64 ± 6 s) of the BTBR males. Similarly the number of chamber entries during social interaction (F (3, 34) = 1.6, *p* = 0.2, pooled X̅ ± SEM = 57 ± 4) and social novelty (F(3, 34) = 1.7, *p* = 0.18, pooled X̅ ± SEM = 46 ± 4) tests did not differ. However, BTBR males given 10 mg/kg CBD dropped half as many feces (3.4 ± 2 vs. 6 to 7 ± 2.2) as other doses (F (3, 34) = 6.19, *p* = 0.002) in the testing chamber during these two sociability tests.

#### Effect of cannabidiol on male C57BL/6J social interaction and novelty preferences

**FIGURE 3:**
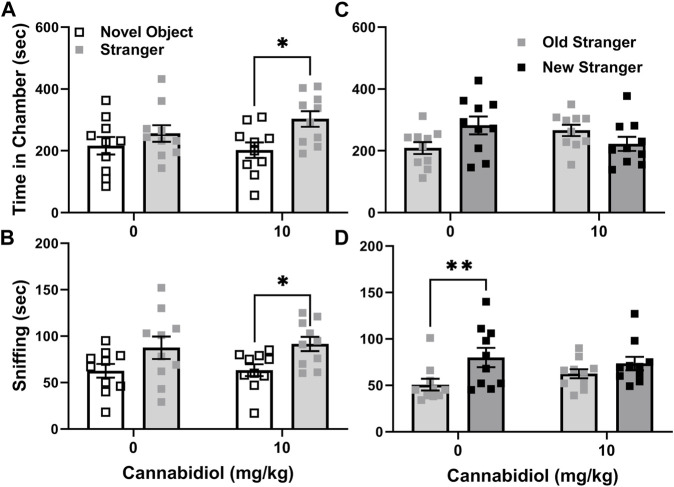
Cannabidiol (10 mg/kg) treatment enhances social interaction preferences but reduces the social novelty sniffing preference of male C57BL/6J mice. **(A)** Cannabidiol and vehicle treated mice did not differ in social interaction preference. However only cannabidiol treated C57BL/6J mice spent significantly more time in chambers with strangers than novel objects (*p < 0.05). **(B)** Cannabidiol treated mice also spent more time sniffing strangers than novel objects (*p = 0.05), while vehicle controls did not. **(C)** In social novelty tests cannabidiol and vehicle treated mice did not differ significantly in preference. Yet vehicle treated mice tended to prefer novel strangers in chamber dwelling time measurements (*p* = 0.06) while cannabidiol treated mice did not. **(D)** By measurements of sniffing time, cannabidiol and vehicle treated mice did not differ in social novelty preference. Within groups cannabidiol treated mice lacked preference for social novelty exhibited by vehicle treated C57BL/6J males (*p < 0.005). Sample sizes were 10 mice per group; data shown are mean ±95% CI, small squares are individual values.

To further assess effects of CBD on social interaction preference at the 10 mg/kg dose, it was tested versus vehicle treatment in adult male C57BL/6J mice, a commonly used strain control for autism-like behaviors of BTBR mice (17). C57BL/6J is the background strain of the SERT knock out mice used in this study (22). In social interaction tests the preference or time difference between stranger and novel object chambers was not affected by 10 mg/kg CBD (t(18) = 0.87, *p* = 0.39). In the REML ANOVA, there was no interaction or CBD effect, but a significant effect of chamber side. This was because CBD treated C57BL/6J mice exhibited preference for interaction as they spent more time in chambers with novel mice than objects (chamber F (1, 36) = 7.1, *p* = 0.01, Sidak’s t(36) = 2.7, *p* = 0.02, Figure 3A), while control mice did not. Measures of sniffing time also revealed no effect of CBD on the differences between stranger mice and novel objects (t(18) = 0.19, *p* = 0.86). The two-way ANOVA again revealed no interaction or CBD effect, but a significant chamber side effect F (1, 36) = 9.35, *p* = 0.005), as CBD treated C57BL/6J sniffed stranger mice more than novel objects (t(36) = 2.3, *p* = 0.05, Figure 3B), while vehicle treated mice did not.

In C57BL/6J social novelty preference tests, the novelty preference, or difference in time with novel stranger versus familiar stranger mice, was unaffected by CBD treatment (t(18) = 1.9, *p* = 0.07). While the REML ANOVA indicated a significant interaction (treatment x chamber F (1, 36) = 6.6, *p* = 0.014), the Sidak’s *post hoc* revealed no effect (t(36) = 2.29, *p* = 0.06) of the 10 mg/kg CBD treatment (Figure 3C). Sniffing preference measures for social novelty tests also revealed no effect of CBD on the difference in time sniffing novel versus familiar strangers (t(18) = 0.17, *p* = 0.12). The REML ANOVA likewise revealed no interaction or effect of CBD, but it showed that vehicle control treated mice preferred new over familiar stranger mice (chamber F (1, 18) = 13.28, *p* = 0.002), as shown in Figure 3D. This significant preference of vehicle control mice for new strangers (Sidak’s t(18) = 3.73, *p* = 0.003), was not seen in the CBD (10 mg/kg) treated C57BL/6J mice (t(18) = 1.41, *p* = 0.3).

There were no differences among vehicle and CBD (10 mg/kg) treated C57BL/6J male mice in time spent in middle chamber during interaction (t(18) = 1.3, *p* = 0.22, X̅ ± SEM = vehicle 128 ± 24 s, CBD treated 95 ± 29 s) or novelty (t(18) = 0.1, *p* = 0.9, X̅ ± SEM = vehicle 109 ± 17 s, CBD treated 111 ± 13 s) tests. Also, the number of chamber entries during interaction (t(18) = 0.8, *p* = 0.39, X̅ ± SEM = vehicle 49 ± 2, CBD treated 45 ± 4) or novelty (t(18) = 0.3, *p* = 0.81, X̅ ± SEM = vehicle 44 ± 4, CBD treated 42 ± 5)) tests did not differ. Also the number of feces dropped (t(18) 0.0, *p* = 0.99) during tests for vehicle controls (5 ± 0.7) and CBD treated (5 ± 0.8) did not differ for C57BL/6J males.

#### Cannabidiol effects on SERT knock-out social preferences

**FIGURE 4:**
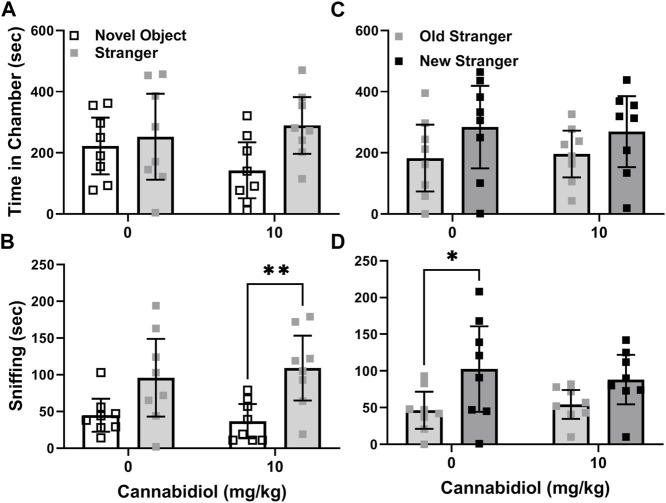
Cannabidiol (10 mg/kg) treatment promotes interaction but not novelty preference in male SERT knock-out mice. **(A)** Cannabidiol and vehicle treated mice did not differ in interaction preference. However, there was a trend (*p* = 0.06) toward cannabidiol treatment increasing SERT knock-out preference for stranger mice over novel objects. **(B)** Cannabidiol treated SERT knock-out mice preferred sniffing strangers versus novel objects (**p < 0.005). **(C)** Cannabidiol treatment had no effect on social novelty preference based on time spent in chambers. **(D)** Vehicle treated mice exhibited social novelty preference in sniffing behavior, while cannabidiol treated mice did not (*p < 0.05). Sample sizes were 8 mice per treatment group; data shown are mean ±95% CI, small squares are individual values.

Serotonin transporter knock-out (SERT KO) mice were previously reported to exhibit deficits in social interaction preference in three chamber choice tests (Moy et al., 2009). For this reason, they were also used to test the effect of the CBD 10 mg/kg dose on social behaviors. In analysis of preference for strangers, no effect of this CBD treatment was evident in the differences in time spent with stranger mice versus novel objects by SERT knock-outs (t(14) = 0.11, *p* = 0.31). REML ANOVA of social interaction tests in the male SERT knock-out mice revealed no significant interaction or CBD treatment effects either. Within groups while social interaction preference by CBD treated SERT knock-out mice appeared to be higher, there was no actual significant difference (chamber F (1, 28) = 3.9, *p* = 0.06) based on time in chambers, as shown in Figure 4A.

In those same social interaction preference tests, CBD (10 mg/kg) had no effect on sniffing time differences between strangers and novel objects (t(14) = 0.66, *p* = 0.5) for the male SERT knock-out mice. Likewise, in the REML ANOVA there was no interaction or CBD effect. However, the chamber side effect was significant ((chamber F (1, 28) = 14.7, *p* = 0.007) in within group comparisons, since the CBD treated SERT knockouts preferred sniffing strangers versus novel objects (Sidak’s t(28) = 3.2, *p* = 0.007, Figure 4B), while the vehicle treated SERT knock-out controls did not (t(28) = 2.2, *p* = 0.06).

Based on SERT knock-out social novelty preference differences, or chamber time with new versus familiar strangers, CBD (10 mg/kg) treatment had no effect (t(14) = 0.29, *p* = 0.78). CBD treatment also had no interaction or main effect on SERT knock-out social novelty chamber side dwelling (chamber F (1, 28) = 3.4, *p* = 0.07, Figure 4C). By measurements of sniffing time differences, the CBD treatment also did not alter SERT knock-out social novelty preference (t(14) = 0.77, *p* = 0.49). In agreement the REML ANOVA indicated no interaction or CBD effect. However, within groups vehicle treated SERT knock-out mice preferred novel strangers (F (1, 14) = 9.8, *p* = 0.007, Sidak’s t(14) = 2.8, *p* = 0.03), while CBD treated ones did not (t(14) = 1.7, *p* = 0.2) (Figure 4D).

There were no differences among vehicle and CBD treated SERT knock-out males in time spent in the middle chamber during interaction (t(14) = 0.75, *p* = 0.46, X̅ ± SEM = 125 ± 33 s control and 172 ± 54s CBD treated) or novelty (t(14) = 0.2, *p* = 0.9, X̅ = 133 ± 67 s control and 134 ± 60 s CBD treated) preference tests. Also chamber entries during interaction (t(14) = 1.8, *p* = 0.09, X̅ ± SEM = 25 ± 4 in controls, 35 ± 3 in CBD treated) or novelty (t(14) = 1.5, *p* = 0.2, X̅ ± SEM = 23 ± 4 in controls, 32 ± 4 in CBD treated) preference tests were not significantly different. Also treatment of SERT knock-outs with CBD (10 mg/kg) did not change the number of feces dropped (t((14) = 1.6, *p* = 0.9, 4.5 ± 1, *p* = 0.87, X̅ ± SEM = 5 ± 1) during tests relative to controls (X̅ ± SEM = 4 ± 1).

### Marble burying

Acute CBD treatments at 0.1–10 mg/kg did not significantly change marble burying (F (3, 34) = 0.4, *p* = 0.7) in male BTBR mice, or at 10 mg/kg in C57BL/6J mice (t(18) = 0.74, *p* = 0.47). Both strains each buried a pooled X̅ ± SEM = 5 ± 3 marbles from the time spanning 70–100 min after injection. SERT knock-out marble burying was not reduced by 10 mg/kg CBD treatment relative to controls (t(14) = 1.5, *p* = 0.2). The SERT knock-outs buried a pooled X̅ ± SEM of 2 ± 0.4 marbles.

### Social dominance

**FIGURE 5:**
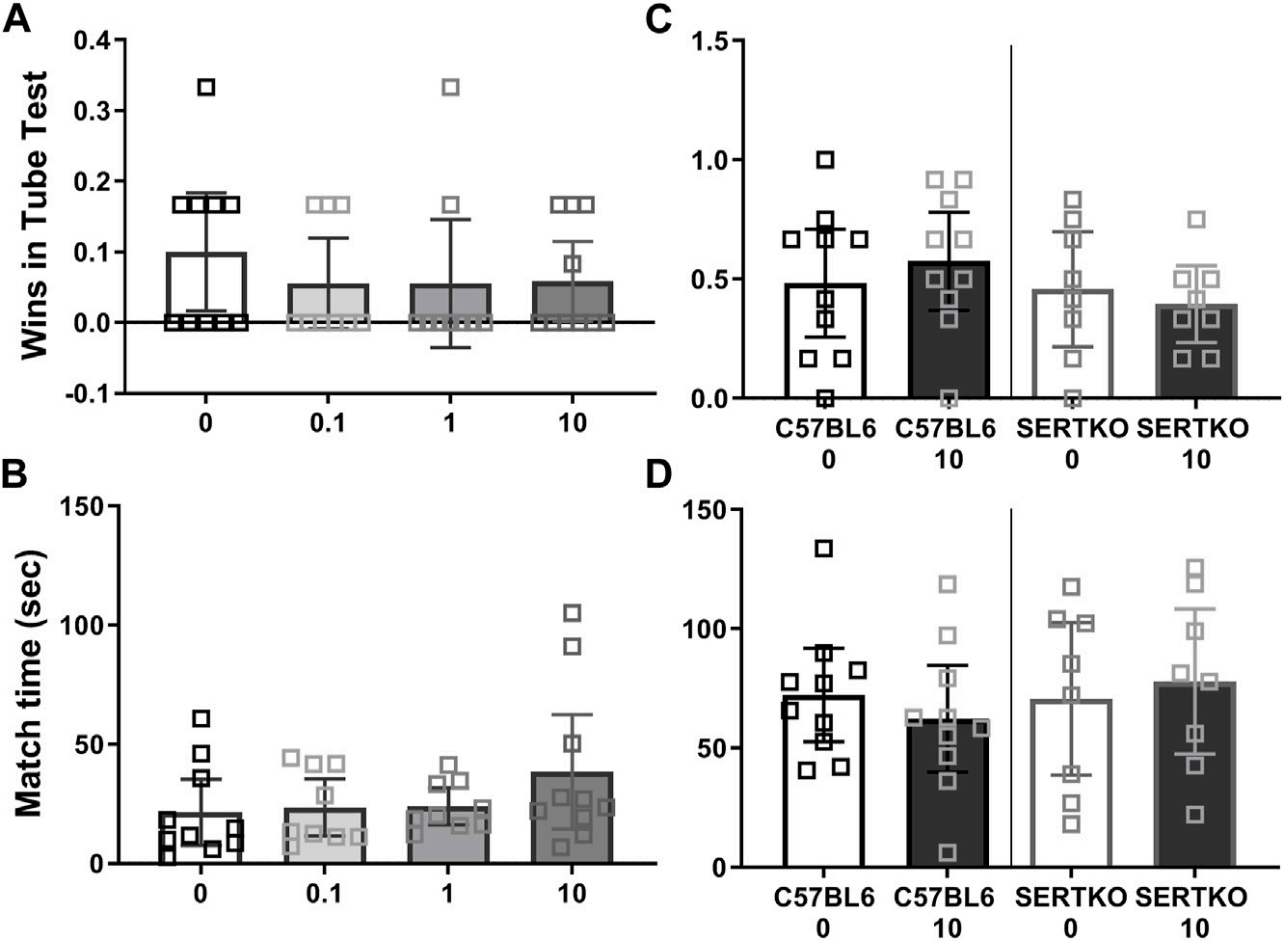
Cannabidiol (10 mg/kg) treatment did not enhance dominance in male mice. Social dominance in male mice was measured starting 2 hours after CBD treatment. We found **(A)** No effect of any CBD dose on BTBR male mouse wins in tube tests. **(B)** The duration of BTBR tube test matches was not impacted by CBD at any dose tested. **(C)** CBD at 10 mg/kg did not alter wins by C57BL/6J or SERT knock-out mice in tube tests. **(D)** CBD at 10 mg/kg did not alter the duration of C57BL/6J or SERT knock-out male mouse tube test matches. Sample sizes are 9–10 for BTBR, 10 for C57BL/6J and 8 for SERT knock-out mice, data shown are mean ±95% CI.

In tube tests no CBD dose tested could increase male BTBR social dominance by measure of wins (Kruskal Wallis test (3, 34) = 1.47, *p* = 0.7), or match duration (F (3, 34) = 1.26, *p* = 0.30, Figures 5A,B). Likewise in C57BL/6J (Mann Whitney U(18) = 41.5, *p* = 0.5) and SERT knock-outs (Mann Whitney U(14) = 27, *p* = 0.6), the 10 mg/kg CBD treatment had no effect on wins (Figure 5C), or match durations (C57BL/6J t(18) = 0.76, *p* = 0.46; SERT knock-outs (t(14) = 0.39, *p* = 0.7, Figure 5D). These tests took place over 100 min after CBD administration.

### Cannabidiol and metabolite levels in serum and brain

**FIGURE 6:**
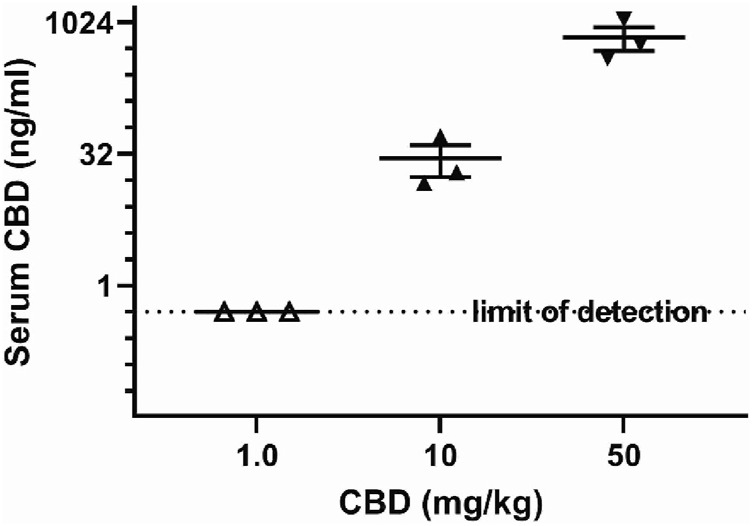
Serum cannabidiol 3 h after intraperitoneal injection at different doses. Samples frozen after collection at −80°C were processed and measured by HPLC with tandem mass spectrographic detection. The limit of detection was 5 ng/mL. Data shown are mean ± SEM, dots shown are individual values.

Serum from 3 male BTBR mice randomly selected for each CBD dose were used to measure CBD and its metabolites 7-OH-CBD, 7-COOH-CBD and 6-α-OH-CBD at 3 h after injection. The detection limit was 0.5 ng/mL and serum CBD was 28.7 ± 14 ng/mL, with lower metabolite levels at 50 mg/kg and at the sociability relevant dose of 10 mg/kg CBD. Levels of CBD are shown in log_2_ scale in Figure 6, and metabolite levels are shown in Table 1. Whole brain levels of CBD for 50 mg/kg at the time of tissue collection after behavior tests were X̅ ± SEM = 3.7 ± 1.2 ng/mg (N = 4 mice), and for 10 mg/kg were 0.1 ± 0.04 ng/mg (N = 5 mice), with a detection limit of 0.05 ng/mg.

**TABLE 1:** Serum levels of cannabidiol and its metabolites 3 h after administration.

CBD dose	CBD (ng/mL)	7-OH-CBD (ng/mL)	7-COOH-CBD (ng/mL)	6-α-OH-CBD (ng/mL)
1 mg/kg	ND	ND	ND	ND
10 mg/kg	29 ± 14	5.4	38.3	9.2
50 mg/kg	692 ± 257	236 ± 83	428 ± 136	142 ± 50

Data are mean ± SEM, unless data from a single sample is reported. Measured from the serum of 3 mice/dose, samples were randomly selected. ND = below the limit of detection. These compounds were also not detectable in serum from vehicle controls.

## Discussion

The main finding of this study is that acute treatment with cannabidiol (CBD) at a dose of 10 mg/kg increased male BTBR mouse social interaction preference relative to the vehicle control treatment, as assessed by difference in time spent in chambers with stranger mice versus novel objects. However, acute CBD (10/mg/kg) effects were not consistent in other measurements of BTBR social interaction preference, such as sniffing times during the social interaction test (see Figure 2C). A strength of our approach was testing acute CBD (10 mg/kg) effects in males from three mouse strains. Two of the strains (BTBR and SERT knock-out) can be considered models of autism and the third is a wild-type background strain for SERT knock-out mice that typically prefers social interactions (16, 17, 19). Even in C57BL/6J mice, within group preference for strangers versus novel objects was more apparent with the CBD treatment (Figures 3A,B). CBD at 10 mg/kg had no effect on time in chambers, but within-group social interaction preference measured by sniffing in SERT knock-out mice was significant only in the CBD treated group (Figure 4B). Taken together these findings, while subtle, indicate that even at a relatively low 10 mg/kg dose CBD has therapeutic potential to enhance social interaction. This is promising since social interaction deficits are the most treatment-resistant autism symptoms (16). Despite the modest number of mice used in this acute treatment pilot study, findings are consistent with those obtained by using CBD in a mouse model of Dravet syndrome (constitutive Scn1a deficient and knock-out or Scn1a+/−) wherein doses of 10 and 20 mg/kg CBD administered an hour before testing also enhanced social interaction in three chamber preference tests (28). The primary goal of this study was to find the lowest dose of CBD that could enhance social interaction preference, and to determine if it could also enhance social dominance or reduce marble burying.

Prior murine studies at higher CBD doses also show its potential to enhance social behaviors. In a mouse model of traumatic brain injury, CBD treatment ameliorated social interaction preference deficits found in the model (29). Likewise in a genetic mouse model of Dravet syndrome (Scn1a +/−) hallmarked by severe epilepsy with co-morbid motor deficits and autism, anxiety and depression an extended CBD treatment of 100 mg/kg, twice daily for 27 days enhanced survival, and dyadic social interaction relative to vehicle controls (30). However, to the best of our knowledge this is the first report of enhanced social preference in three chamber social interaction and novelty preference tests by acute CBD treatment in BTBR or SERT knock-out mice. The vehicle-treated (10% methanol) C57BL/6J males exhibited less preference for interaction than we have seen in prior studies without alcohol administration (26). This effect may account for the unexpected improvement in their social interaction with 10 mg/kg CBD treatment (Figures 3A,B).

Social novelty preference was not enhanced by this regimen of acute CBD treatments in any strain or line of mice examined in the present study. The social novelty preference tests started 60 min and ended 70 min after CBD injection. This appears to be a peak time for CBD to impact mouse social interaction preference behaviors, consistent with findings 1 hour after treatment in other strains, however CBD effects on social novelty preference was not previously examined in mice (28). However, in rats CBD treatments of 12 and 30 mg/kg impaired social recognition memory in a parallel test (31). Intriguingly, in the present study it also appeared that in C57BL/6J as well as in SERT mice sniffing of novel stranger mice was somewhat dampened. However, since the interaction was not significant and the study may have been underpowered, these findings should be interpreted cautiously.

Marble burying behavior was unaffected by 10 mg/kg CBD in the present study at 70–100 min after administration. Prior reports indicate that a dose of 30–120 mg/kg CBD can suppress marble burying in mice acutely or when administered for several days (32). The 10 mg/kg CBD dose administered in the present study may have been insufficient to reach the threshold required to reduce marble burying. Indeed the 4 mice administered CBD at a dose of 50 mg/kg in 50% methanol vehicle did not bury any marbles or back out of the tube test, but since their locomotor and/or exploratory activity appeared to be inhibited, and this effect could not be teased apart from the higher concentration of methanol required to achieve this dose we did not pursue any higher CBD doses since it fell outside of the scope of this pilot study.

This study has three major limitations, the first is the inability to perform additional experiments due to the discontinuation of the commercial formulation of CBD in methanol used. Our use of 10% methanol as a solvent was necessitated by initial use of the United States Drug Enforcement Administration (USDEA) exempt preparation of CBD, of >98 purity that was available at the time of the study from Cayman Chemical Co. (Catalog #90081, discontinued). This formulation limited the present study to acute treatments because of the methanol solvent, and subsequently it became unavailable. The equivalent CBD reference is now sold only in an acetone solvent, so we were unable to increase our sample size or expand the study. For future studies, which should include tests of these behaviors after chronic CBD administration, a different solvent, such as 1:1:18 mixture of Tween 80, propylene glycol, and physiologic saline (33), or a 1:1:18 mixture of saline, ethanol, and emulphor (14) will provide more flexibility and stability.

The second major limitation of this study is the lack of female mouse subjects. Our justification for excluding females from this pilot study was that ASD is 4 times more common in males than in females (16). However, sex differences in autism incidence and manifestation deserve greater attention and scrutiny in future clinical and preclinical studies. Clinical manifestation of ASD can be more difficult to detect in females due to greater female conformance to gender stereotypes, gender-specific narrow interests that are more socially oriented, and greater symptom camouflaging by girls with ASD (34–36). Indeed, ASD diagnoses are typically made later in females than in males (34), and the true male/female ratio for ASD may be closer to 3:1 (37). In mice male-female comparisons have been made for autism-relevant behaviors, and in many of them the social behavioral phenotypes of both sexes is similar (16, 38). Even in response to CBD treatment, male and female behaviors were similar in the Dravet syndrome mouse model (28). However, it remains to be demonstrated if such effects are more widely generalizable in the BTBR or SERT knockout mouse or in other ASD models wherein females were not widely studied.

The third major limitation of this preliminary study is that the mechanism of action of CBD to enhance the social behavior of BTBR mice was not elucidated. CBD has more than 65 potential therapeutic targets, and comprehensive studies to discover CBDs role in shaping behaviors through them are just beginning to unfold (1, 39). Strengths of this study include the use of multiple mouse models of ASD, a variety of behavioral tests, and measurement by mass spectrometry of serum and brain homogenate CBD and our attempt to also measure the metabolite levels. The pharmacokinetics of acute CBD administration have yet to be established in the BTBR, C57BL/6J or SERT knock-out mice, and for this reason it is unclear how long sociability benefits may last. The 10 mg/kg dose of CBD was still detectable in the BTBR brain 3 h after injection, but the pharmacological targets producing the enhanced sociability remain unclear. Future studies are needed to investigate the acute CBD behavioral time course and brain regions for each type of behavior test in these specific mouse strains and lines. Chronic treatment studies are critically needed to discover other potentially beneficial behavioral effects and side effects.

We did not find any impact of CBD treatment up to 10 mg/kg on social dominance or marble burying. One possibility is that the effects of CBD acutely administered at 10 mg/kg may have worn off before these tests were performed. Alternatively, the sequence of prior testing may have dampened the effects of CBD treatment in subsequent tests. Randomized controlled clinical trials, are now underway for use of CBD, mixtures of CBD and THC, and structurally related compounds such as cannabidivarin to treat autism symptoms (6). However, isolating the mechanism(s) of action responsible for enhancing social interaction preference will still require further preclinical studies in animal models, as such efforts may yield more selective treatments for social behavior deficits.

CBD has many different pharmacological effects, and potential physiological mediators (1, 39). Based on previously established affinities, CBD administered to mice at 10 mg/kg is unlikely to bind to and compete with CB_1_ or CB_2_ receptors directly (10). Interestingly, the effects of CBD (15 mg/kg) to attenuate aggressive attacks of strangers by socially isolated male mice was blocked by CB_1_ and 5-HT_1A_ antagonists AM251 and WAY100635, which demonstrated the role of these receptors in CBD modulation of aggressive behavior (15). When CBD treatment effects were tested in dyadic social interactions with an unrestrained stranger mouse in an MK-801 model of schizophrenia, they found the 5-HT_1A_ antagonist WAY100635, but not CB_1_ antagonist AM251 or CB_2_ receptor antagonist AM630 blocked the ability of CBD to enhance social interactions (40). Thus 5-HT_1A_ receptor modulation may be a key mediator of social boldness or dominance, and the sensitivity of BTBR mice to CBD may differ from C57BL/6J mice, since we previously found inherent upregulation of 5-HT_1A_ as well as CB_1_ receptor expression and G-protein coupling capacity in this strain (26). Perhaps 5-HT_1A_ receptor impairment may account in part for why BTBR social dominance was so low in BTBR mice (Figure 5). Indirect actions of CBD at 5-HT_1A_ receptors is consistent with our prior discovery that the partial 5-HT_1A_ agonist buspirone (2 mg/kg) acutely enhanced social interaction in 3 chamber tests of male BTBR mice (41). However direct CBD binding to 5-HT_1A_ is unlikely to be a mediator of these effects, based on CBD’s relatively low affinity for this serotonin receptor subtype (10).

We also see in constitutive SERT knock-out mice, which have elevated extracellular 5-HT levels, that acute CBD treatment modestly enhanced social sniffing (Figure 4B). This outcome could be consistent with a corrective, albeit indirect, action of CBD at 5-HT_1A_ receptors, given that reductions only in 5-HT_1A_ autoreceptor density in the dorsal raphe were found in male SERT knock-out mice (42), or that postsynaptic 5-HT_1A_ feedback inhibition is enhanced in SERT knockout mice (43). For example, treatment with the 5-HT1A antagonist WAY 100635 reduced anxiety-like traits in SERT knock-out, but not wild-type mice (44). CBD modulation of 5-HT_1A_ receptor properties in constitutive SERT knock-out mice is worthy of further study, as it may produce compensatory modifications in the sensitivity of this receptor.

The atypical cannabinoid receptor GPR55 is another promising therapeutic target of CBD, it occurs in many brain areas including hippocampus, cortex, striatum, and cerebellum (45), and in the blood-brain barrier (46). GPR55 is involved in spatial learning and memory (45), and its expression is doubled in Rett’s syndrome mice exhibiting deficits in social behavior (47). A structurally related cannabinoid to CBD, cannabidivarin (1), acted as an antagonist at GPR55, and it enhanced social behavior in the Rett’s syndrome mice (47). However, properties of CBD at GPR55 remain unclear. Prior reports indicate CBD may be a GPR55 agonist, however, a modified form of CBD examined was not selective as a GPR55 agonist, as evidenced by its effects at a 1 µM dose in GPR55 knock-out mice (48). Now that more selective GPR55 agonists such as O-1602 and CID2440433 are available, their use to study CBD action at GPR55 warrants further investigation of GPR55 as a therapeutic target for social deficits.

Additionally, adenosine A_2A_ receptors are involved in CBD action to blunt cognitive impairing effects of THC in hippocampus, and CB1 receptors dimerize with A_2A_ receptors *in vivo in* hippocampal CA1, interacting in a way that may mediate such effects (49). Other promising CBD targets that may serve modulate social behaviors as well as sensory input are transient receptor potential (TRP) and other ion channels, several of which are reported to be dysfunctional in some forms of autism (50). BKCa channels (51), as well as TRP channels, especially TRPV3 and TRPV4 are prominent examples of ion channels that CBD is known to target (12, 52–54). At many TRPVs CBD was found to act as an agonist (12), and this property may be counterproductive for its use as an autism treatment, unless the TRPVs are desensitized by such treatments, as occurs with TRPV1 (55). For example, in the Shank3 mutation mouse model of ASD, social behavior was impaired and TRPV4 upregulated, so infusion of HC-067047, a TRP4 antagonist, into the nucleus accumbens increased mouse social interaction preference (56).

Finally, in addition to known receptor targets, CBD may have other indirect and unanticipated effects. For example, two-week treatment with CBD produced epigenetic changes in the mouse hippocampus, which overall favored hypomethylation (57). Studies in animals raised concerns that orally administered CBD could be converted to THC during the digestive process, but this does not appear to occur in humans (58). CBD may also inhibit uptake of or catabolism of the endogenous cannabinoid anandamide among others (59). In adult men with and without autism, CBD administration paired with magnetic resonance spectroscopy revealed that CBD treatment modulated glutamate and γ aminobutyric acid systems differently (60). This may stem from CBD action at transient receptor potential cation channel vanilloid receptor 1 (TRPV1), which it activates and de-sensitizes (55). These same researchers demonstrated TRPV1 is found near high density glutamate expressing areas of the hippocampal cornu ammonis 1 (CA1), which is potentially involved in shaping social interaction and novelty preference (26). But most likely of all mechanisms based on binding affinity in mice, CBD competitively inhibits fatty acid amide hydrolase (FAAH), the main enzyme breaking down endocannabinoids, thereby increasing brain levels of anandamide and other endocannabinoids (13). This could be the primary mechanism to acutely increase sociability, as we saw in our mice. However, there is controversy regarding the ability of CBD to reduce human FAAH activity (59). Overall, among the potential CBD mediated mechanisms, the ones activated by 10–20 mg/kg doses are of greatest interest based on the present study, and the prior findings of Kaplan et al. (28).

Due to changes in laws regarding CBD use, it is increasingly available from dispensaries, and is frequently used without physician consultation (61). However, unregulated CBD products are often unrefined, and aside from reporting CBD/THC ratio, their potential content of up to 118 other *C. sativa* compounds are frequently unknown (1). Heedless of lack of preclinical or clinical research evidence, CBD is perceived to be not only beneficial for alleviating pain, but also as a potential treatment of psychiatric disorders by the public (62). While mouse studies of CBD analgesic properties are more numerous and have included pharmacodynamics of oral administration (63), our knowledge of CBD effects on these and other social behaviors still lags. This is compounded by the fact that unregulated CBD products are less expensive than prescription grade purified CBD (64). However, use of unregulated CBD products is not advisable, especially if the purity and dose of such products are not strictly controlled. Even a non-intoxicating compound such as CBD may have unforeseen adverse effects in juveniles, given the potential susceptibility of the developing cannabinoid system to disruption, and its critical role in shaping social behaviors (65–69). Therefore, the need for further preclinical research on the therapeutic potential of CBD for social behavior deficits and expanding the knowledge of its underlying mechanisms is paramount to developing targeted therapies for autism and related disorders.

## Conclusion

In sum, this study demonstrates that 10 mg/kg CBD treatment was able to acutely enhance social interaction preference, albeit incompletely for some and in slightly different ways, in three kinds of adult male mice. Future studies should include acute CBD treatments in juvenile, adolescent and female mice, as well as chronic administration for further assessment of the potential of purified CBD as an autism treatment and finding mechanism(s) underlying CBD’s sociability enhancing properties. Further preclinical studies in laboratory animals are essential to identify which pharmacological action(s) of acute CBD treatment are responsible for the improvements in social interaction preference found in this study.

## Data Availability

The original contributions presented in the study are included in the article/supplementary material, further inquiries can be directed to the corresponding author.
